# Comparison of Two Genotyping Methods for Distinguishing Recrudescence from Reinfection in Antimalarial Drug Efficacy/Effectiveness Trials

**DOI:** 10.4269/ajtmh.18-0002

**Published:** 2018-05-21

**Authors:** Joseph R. M. Fulakeza, Rachel L. Banda, Trancizeo R. Lipenga, Dianne J. Terlouw, Standwell C. Nkhoma, Eva Maria Hodel

**Affiliations:** 1 University of Malawi, College of Medicine, Blantyre, Malawi;; 2 Malawi-Liverpool-Wellcome Trust Clinical Research Programme, Blantyre, Malawi;; 3 Liverpool School of Tropical Medicine, Liverpool, United Kingdom

## Abstract

Genotyping of allelic variants of *Plasmodium falciparum* merozoite surface proteins 1 and 2 (*msp-1* and *msp-2*), and the glutamate-rich protein is the gold standard for distinguishing reinfections from recrudescences in antimalarial drug trials. We compared performance of the recently developed 24-single-nucleotide polymorphism (SNP) Barcoding Assay against *msp-1* and *msp-2* genotyping in a cluster-randomized effectiveness trial of artemether–lumefantrine and dihydroartemisinin–piperaquine in Malawi. Rates of recrudescence and reinfection estimated by the two methods did not differ significantly (Fisher’s exact test; *P* = 0.887 and *P* = 0.768, respectively). There was a strong agreement between the two methods in predicting treatment outcomes and resolving the genetic complexity of malaria infections in this setting. These results support the use of this SNP assay as an alternative method for correcting antimalarial efficacy/effectiveness data.

## INTRODUCTION

In areas of intense malaria transmission, drug-treated malaria patients are at high risk of reinfection during long follow-up post-treatment. Without genotyping pretreatment and post-treatment parasites, it is difficult to resolve whether parasites persisting after therapy are due to treatment failure (recrudescence) or a new infection (reinfection) and to provide the true risk of treatment failure in the population.^1^

Genotyping of allelic variants of *Plasmodium falciparum* merozoite surface proteins 1 and 2 (*msp-1* and *msp-2*), and glutamate-rich protein is the recommended genotyping method.^1,2^ However, it is labor intensive, has low discriminatory power, and produces results that are often ambiguous to interpret and reproduce between laboratories.^3^ Microsatellite genotyping is an alternative approach.^4–6^ However, the lack of capillary sequencers to amplify and score microsatellites has hampered its wide use. The 24-single-nucleotide polymorphism (SNP) Barcoding Assay has shown great potential^7^ but requires expensive reagents and real-time polymerase chain reaction (PCR) instruments. We compared the performance of the 24-SNP Barcoding Assay and *msp-1* and *msp-2* genotyping in an effectiveness trial.

## METHODS

This study was part of a trial exploring neuro-ototoxic adverse effects in children repeatedly treated with artemisinin-based combination therapies (NCT01038063). Ethical approvals were obtained from Liverpool School of Tropical Medicine Research Ethics Committee (Protocol 09.07), University of Malawi College of Medicine Research and Ethics Committee (Protocol P.10/08/707), and Malawi’s Pharmacy, Medicines and Poisons Board (Protocol PMPB/CTRC/III/1211200904).

Children with uncomplicated malaria were randomized to receive artemether–lumefantrine (AL) or dihydroartemisinin–piperaquine (DHA-PPQ) and followed up for 42 days. A filter paper blood sample was collected before treatment and 42 days posttreatment regardless of day 42 slide positivity.

To determine if a child had recurrent parasitemia on day 42, parasite DNA was extracted from d0 and d42 samples using DNA Mini Kits (Qiagen, Manchester, United Kingdom) and genotyped using the 24-SNP Barcoding Assay, and *msp-1* and *msp-2* genotyping as previously described.^2,7^ Investigators genotyping samples were blinded to d42 slide positivity. Infections with ≥ 2 and ≤ 1 heterozygous SNPs were classified as multiple- and single-haplotype infections, respectively.^8^ We performed a loci resampling analysis in GenClone v.2.0^9^ to determine the minimum number of SNPs required to capture full haplotypic diversity amongst single-haplotype infections sampled.

Recurrent parasitemia was considered a reinfection if d0 and d42 parasites were genetically distinguishable; otherwise, it was deemed a recrudescence. All proportions and their binomial exact 95% confidence intervals (CIs) were computed using Stata version 11.0 (College Station, TX).

## RESULTS AND DISCUSSION

We evaluated 109 pairs of filter paper blood samples collected on days 0 and 42. Of these, 65% (*N* = 71) showed no detectable parasite DNA on d42, whereas 38 had recurrent d42 parasitemia. Detailed effectiveness data for the trial will be presented elsewhere (D. J. Terlouw et al., unpublished data). Genotype data and treatment outcomes for 38 patients with recurrent parasitemia are shown in Supplemental Tables 1 and 2, whereas genotype data for 71 patients with no detectable parasite DNA on d42 are shown in Supplemental Tables 3 and 4. A sample size of 38 recurrent infections allows us to detect a 34% difference in rates of reinfection estimated by the two methods with 80% power and 95% CI. Repeat *msp-1* and *msp-2* genotyping was performed on ∼20% of samples because of contamination in the negative control or failure to amplify some loci during the initial genotyping attempt. However, genotyping failure rate for the 24-SNP Barcoding Assay was low with > 95% of SNP assays yielding data at the first genotyping attempt and < 5% allele drop out per sample.

**Figure 1.**
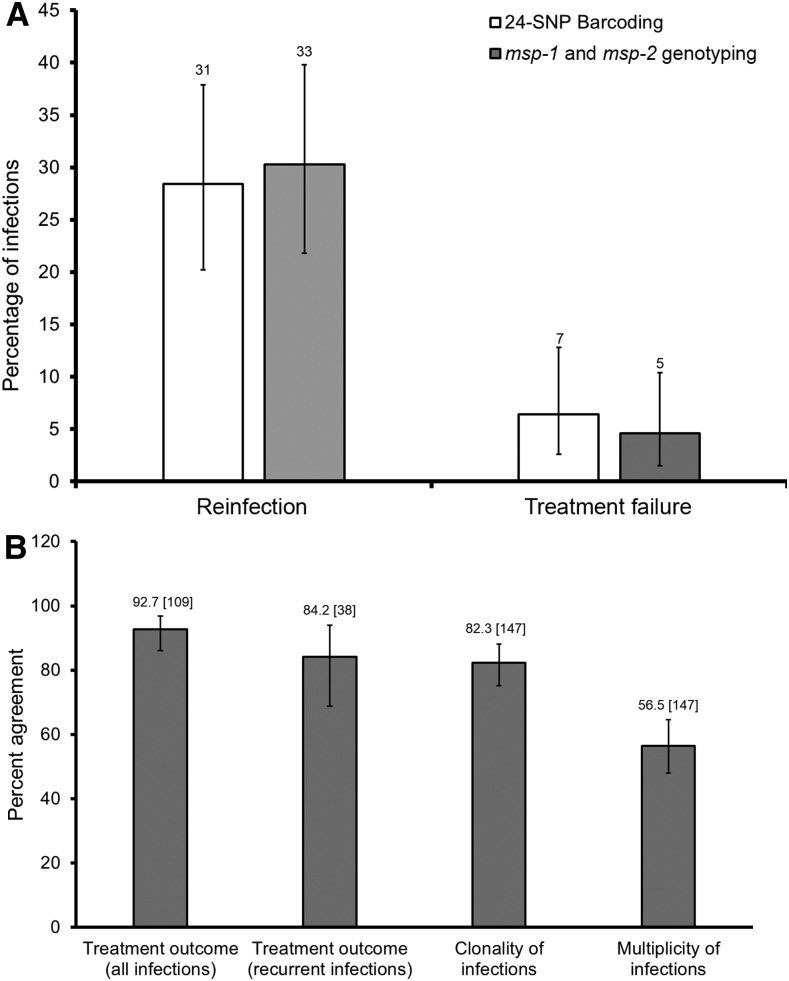
Comparison of two genotyping methods. (**A**) Rates of reinfection and recrudescence estimated by the 24-single-nucleotide polymorphism (SNP) Barcoding Assay and merozoite surface proteins 1 and 2 (*msp-1* and *msp-2*) genotyping. The number on top of each bar represents number of patients with a defined treatment outcome out of 109 patients evaluated. Rates of reinfection and recrudescence estimated by the two methods were similar (Fisher’s exact test; *P* = 0.887 and *P* = 0.768, respectively). (**B**) Agreement between methods in determining treatment outcomes, infection clonality, and multiplicity of infection. Figures on top of each bar are percentages of concordant samples out of all samples analyzed in square brackets. Multiplicity of infection was determined from SNP data of each sample using complexity of infection likelihood (COIL)^14^ and from *msp-1* and *msp-2* data as the highest number of alleles observed at the most diverse locus. In both **A** and **B**, error bars are binomial exact 95% confidence intervals.

Rates of reinfection and treatment failure did not differ significantly between methods (Fisher’s exact test; *P* = 0.887 and *P* = 0.768, respectively) (Figure 1A). There was a strong concordance between the two methods in predicting treatment responses among all the 109 patients evaluated and in 38 patients with recurrent d42 parasitemia (Figure 1B). There was also a strong agreement between the two methods in determining the clonality of parasite samples (whether a sample is monoclonal or multiclonal) (Figure 1B). The proportion of multiclonal samples was similar between methods (Supplemental Figure 1). Relationships among 62 monoclonal samples identified using the 24-SNP Barcoding Assay are shown in the phylogenetic tree (Supplemental Figure 2). We observed a modest concordance of 56.5% (binomial exact 95% CI: 48.0–64.6) between the two methods in estimating the multiplicity of infection for individual samples (Figure 1B). This presumably reflects subtle differences in the resolution power of the two assays. Treatment failure rate was 6.4% by the 24-SNP Barcoding Assay and 4.6% by *msp-1* and *msp-2* genotyping (*P* = 0.768). The small discrepancy between recrudescence rates estimated by the two methods resulted from classifying two recurrent infections either as treatment failures when using SNP genotyping or as as reinfections when using *msp-1* and *msp-2* genotyping (Supplemental Tables 1 and 2). Treatment failures observed may be explained by nonadherence, pharmacokinetic variations, parasite resistance, and/or drug loss through vomiting. Study participants were given a full course of AL or DHA-PPQ with only the first dose given under supervision. This may promote noncompliance but accurately represents how drugs might be used in the community. In a previous study, 79% and 88% of AL- and DHA-PPQ–treated patients complied with recommended drug dosing schedules, respectively.^10^

High rates of reinfection are of concern. Both genotyping methods showed that ∼30% of children treated for malaria are reinfected within 42 days post-treatment. This finding indicates that the intensity of transmission is very high. Compared with DHA-PPQ, AL is associated with higher risk of recurrent parasitemia^11,12^ attributable to shorter elimination half-life of the partner drug, lumefantrine. However, an ACT such as DHA-PPQ, with a long elimination half-life of the partner drug, may still fail to protect against reinfections if overwhelmed by intense transmission levels.^11^ To help reduce malaria transmission, new transmission reduction strategies such as mass drug administration, focal screening and treatment, or mass screening and treatment should be considered.^13^

**Figure 2.**
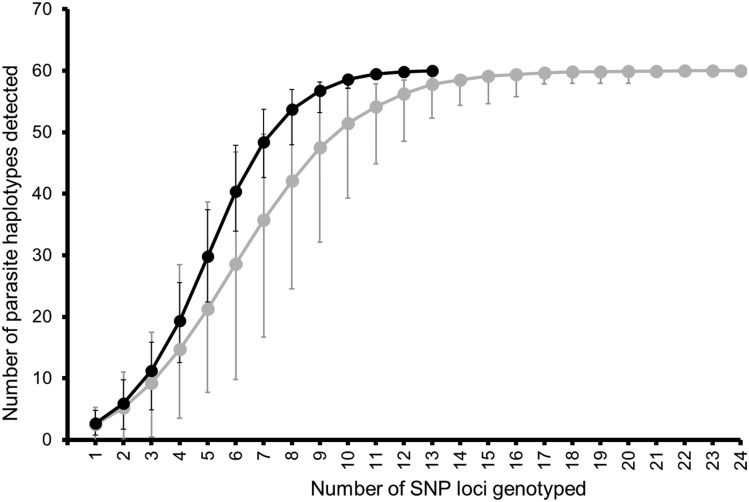
Resolution power of the 24-single-nucleotide polymorphism (SNP) Barcoding Assay inferred from SNP resampling. The gray line shows maximum haplotype diversity captured when all 24 SNPs are used to characterize diversity, whereas the black line indicates diversity identified when only SNPs with a high minor allele frequency (≥ 0.30) are used. Error bars are 95% confidence intervals for the mean number of parasite haplotypes identified. Diversity plateaus after 17 and 12 loci if all 24 SNPs and SNPs with high minor allele frequency are used to genotype samples, respectively, indicating the assay’s sufficient discriminatory power.

Our findings clearly demonstrate that the 24-SNP Barcoding Assay performs as well as *msp-1* and *msp-2* genotyping. The main advantage of *msp-1* and *msp-2* genotyping is its low cost. We estimate that genotyping costs $11.45/sample versus $3.60/sample for the 24-SNP Barcoding Assay and *msp-1* and *msp-2* genotyping, respectively. Unlike the 24-SNP Barcoding Assay that relies on expensive real-time PCR instruments, *msp-1* and *msp-2* genotyping uses relatively inexpensive and common laboratory equipment such as gel electrophoresis equipment and ultra-violet (UV) transilluminators to genotype samples. Nonetheless, inherent limitations of *msp-1* and *msp-2* genotyping outweigh its low-cost attractiveness. This method is extremely labor intensive, prone to contamination, has limited resolution power, and generates data that are often ambiguous to interpret and reproduce between different laboratories because of dependency on visual interpretation of allele migration patterns on agarose gels. In contrast, the 24-SNP Barcoding Assay is less labor intensive, has better resolution power, and generates data that are easy to score and reproduce between laboratories. The 24-SNP Barcoding Assay has better discriminatory power because it interrogates 24 highly polymorphic SNPs rather than two *msp-1* and *msp-2* loci. Because of its excellent attributes, the 24-SNP Barcoding Assay should be adopted as an alternative genotyping method. However, high cost could derail its adoption. We investigated whether an abbreviated SNP set with fewer SNPs could equally identify all parasite haplotypes as 24 SNPs. Our results indicate that 17 SNPs, irrespective of their minor allele frequencies within the 62 single-haplotype infections identified, can reliably capture all parasite haplotypes identified by 24 SNPs (Figure 2, Supplemental Table 1). Our data also indicate that if SNPs with a high minor allele frequency (≥ 0.30) are selected, only 12 of these are required to identify all parasite haplotypes (Figure 2, Supplemental Table 1). It would cost $5.73 to genotype a single sample using the abbreviated SNP assay. Reduction in cost and availability of real-time instruments in most countries make the abbreviated SNP assay attractive and feasible to adopt.

## CONCLUSION

Our results demonstrate that the 24-SNP Barcoding Assay performs as well as *msp-1* and *msp-2* genotyping and should be adopted as an alternative method for PCR adjustment of antimalarial effectiveness/efficacy data. Resource-constrained laboratories should consider deploying an abbreviated SNP assay comprising 12 SNPs with high minor allele frequency to reduce genotyping costs while maintaining high assay resolution. Each continent must identify SNPs with high minor allele frequency to select informative SNPs.

## Supplementary Material

Supplemental reference, figures, and tables
