# Supplementary material for: Comparison of Two Genotyping Methods for Distinguishing Recrudescence from Reinfection in Antimalarial Drug Efficacy/Effectiveness Trials
Source: Am J Trop Med Hyg. 2018 May 21;99(1):84–6. doi: 10.4269/ajtmh.18-0002 (PMC6085787; doi:10.4269/ajtmh.18-0002)
Supplement: Supplementary file 1 [file tpmd180002.SD1.pdf]

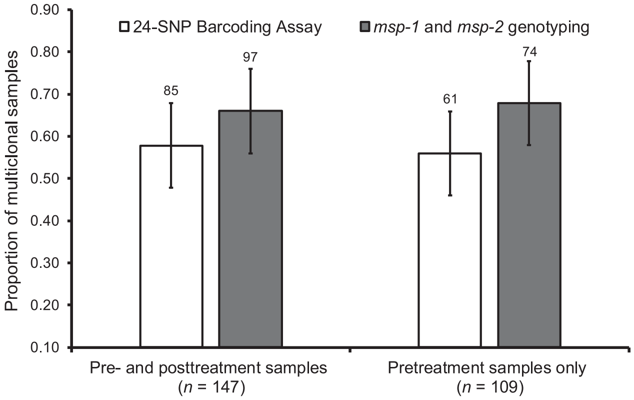

Supplemental Figure 1. Proportion of multiclonal samples estimated by the 24-single-nucleotide polymorphism (SNP) Barcoding Assay compared with that determined by merozoite surface proteins 1 and 2 (*msp-1* and *msp-2*) genotyping. The proportion of multiclonal samples in a combined set of pretreatment and posttreatment samples ($N = 147$) and in pretreatment samples alone ($N = 109$) did not differ significantly between the two methods (Fisher's exact test; $P = 0.186$ and $P = 0.094$, respectively). Error bars are binomial exact 95% confidence intervals for the proportion of multiclonal samples. The number on top of each bar represents the number of multiclonal samples detected by each of the two genotyping methods. For example, the proportion of pretreatment samples deemed to be multiclonal by the 24-SNP Barcoding Assay equals 61/109 = 0.560.

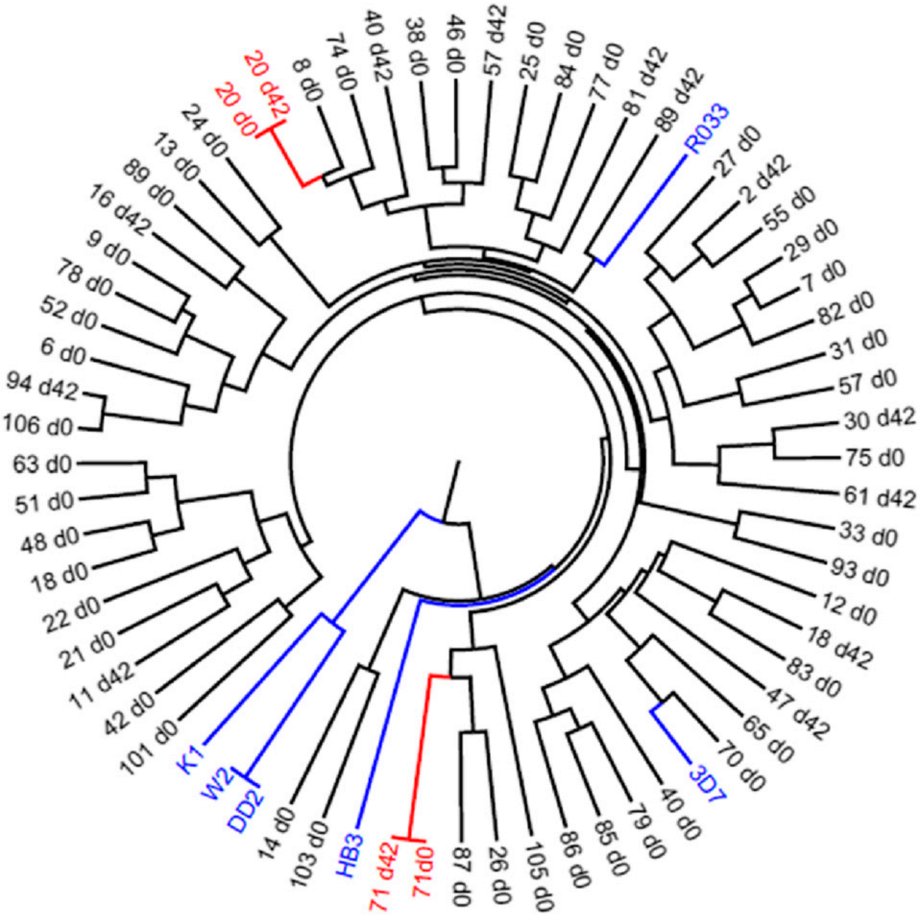

Supplemental Figure 2. Unweighted pair group method with arithmetic mean (UPGMA) tree showing relationships between parasite haplotypes identified by the 24-single-nucleotide polymorphism (SNP) Molecular Barcode Assay. We computed the proportion of SNP alleles shared (ps) between all pairwise comparisons of single-haplotype infections sampled and clustered infections on the UPGMA tree based on the genetic distance metric, 1-ps, using the Phylogeny Inference Package (PHYLIP).[18] Only data for single-haplotype parasite infections are shown because allele-sharing can be unambiguously computed. Pretreatment episodes of parasitemia in patients 20 and 71 (i.e., 20d0 and 71d0) have the same parasite DNA fingerprint as their respective posttreatment, episodes 20d42 and 71d42. Therefore, recurrent episodes of parasitemia in patients 20 and 71 are treatment failures. On the other hand, posttreatment episodes of parasitemia in patients 18 and 40 (i.e., 18d42 and 40d42) are genetically different from pretreatment episodes (18d0 and 40d0). These are a classical case of reinfection.

SUPPLEMENTAL REFERENCE

18. Felsenstein J, 1993. *PHYLIP (Phylogeny Inference Package) v.3.57*. Distributed by the Author. Seattle, WA: Department of Genetics, University of Washington.
